# Luminescence Studies and Judd–Ofelt Analysis on SiO_2_@LaPO_4_:Eu@SiO_2_ Submicro-spheres with Different Size of Intermediate Shells

**DOI:** 10.1038/s41598-019-49323-6

**Published:** 2019-09-10

**Authors:** Xiaowei Zhu, Kuisuo Yang, Anping Wu, He Bai, Jinrong Bao, Yan Qiao, Yunjiang Yang, Wenxian Li, Ying Liu

**Affiliations:** 1 College of Pharmacology, Inner Mongolia Medical University, Hohhot, 010059 China; 2 Inner Mongolia Key Laboratory of Chemistry and Physics of Rare Earth Materials, College of Chemistry and Chemical Engineering, Inner Mongolia University, Hohhot, 010021 China

**Keywords:** Chemistry, Inorganic chemistry, Optics and photonics, Optical materials and structures, Materials for optics

## Abstract

The novel submicro-spheres SiO_2_@LaPO_4_:Eu@SiO_2_ with core-shell-shell structures were prepared by connecting the SiO_2_ submicro-spheres and the rare earth ions through an organosilane HOOCC_6_H_4_N(CONH(CH_2_)_3_Si(OCH_2_CH_3_)_3_ (MABA-Si). The as-prepared products were characterized by X-ray diffraction (XRD), scanning electron microscopy (SEM), transmission electron microscopy (TEM), X-ray photoelectron spectroscopy (XPS), and infrared spectroscopy (IR). It is found that the intermediate shell of the submicro-spheres was composed by LaPO_4_:Eu nanoparticles with the size of about 4, 5–7, or 15–34 nm. A possible formation mechanism for the SiO_2_@LaPO_4_:Eu@SiO_2_ submicro-spheres has been proposed. The dependence of the photoluminescence intensity on the size of the LaPO_4_:Eu nanoparticles has been investigated. The intensity ratios of electrical dipole transition ^5^D_0_ → ^7^F_2_ to magnetic dipole transition ^5^D_0_ → ^7^F_1_ of Eu^3+^ ions were increased with decreasing the size of LaPO_4_:Eu nanoparticles. According to the Judd-Ofelt (J-O) theory, when the size of LaPO_4_:Eu nanoparticles was about 4, 5–7 and 15–34 nm, the calculated J-O parameter Ω_2_ (optical transition intensity parameter) was 2.30 × 10^−20^, 1.80 × 10^−20^ and 1.20 × 10^−20^, respectively. The increase of Ω_2_ indicates that the symmetry of Eu^3+^ in the LaPO_4_ lattice was gradually reduced. The photoluminescence intensity of the SiO_2_@LaPO_4_:Eu@SiO_2_ submicro-spheres was unquenched in aqueous solution even after 15 days.

## Introduction

Recently, core-shell structured nanomaterials have attracted the researcher’s attention because of their multifunctional cores and shells. Since the structure and the composition can be easily modified by a controllable way, the optical, thermal, and catalytic properties of the core-shell nanomaterials might be tailored and be used in various fields^1–6^. Silica is often used as a coating material due to its high chemical stability and good physico-chemical properties^7,8^. In addition, if the core-shell nanomaterials were coated on the silica core, the overall cost of the luminescent materials might be greatly reduced. Moreover, the photoluminescence intensity of phosphor can be significantly enhanced by these SiO_2_ core-shell materials: silica shell can strengthen the stability of materials to protect the core materials from dissolution or hydrolysis, while the Si–OH groups bonds can make the SiO_2_ easily bond with bio-macromolecule and then improve its biocompatibility. Therefore, the core-shell nanomaterials are good candidates for biological applications^9,10^. To date, many methods have been studied on the controlled fabrication of SiO_2_ core-shell luminescent materials. For example, Ansari *et al*. have synthesized silica-coated luminescent Y_2_O_3_:Eu nanoparticles by using an urea-based decomposition process. The mesoporous SiO_2_ layer played an important role in perfecting the Y_2_O_3_:Eu nanoparticles^11^. In addition, the SiO_2_@TiO_2_:Sm^3+^ hybrid materials have been prepared by a solvothermal coating method. It was found that the SiO_2_ core inhibited the growth of the TiO_2_ particles and reduced its aggregation and poor dispersity^12^. Tong *et al*. have synthesized Fe_3_O_4_@SiO_2_@Y_2_O_3_:Eu^3+^ composites, which exhibited ferromagnetic behavior and strong luminescence intensity^13^. Atabaev *et al*. have reported that the SiO_2_@Y_2_O_3_:Eu^3+^, Co^2+^ core-shell phosphor composites might be used in the biomedical applications because of their magnetic and luminescent properties^14^. The CePO_4_:Tb@LaPO_4_@SiO_2_ composites could also be synthesized by a co-precipitation process. The silica shell improved the solubility and colloidal stability of CePO_4_:Tb@LaPO_4_ in the solvent^15^. Secu *et al*. have found that the BaFBr:Er^3+^@SiO_2_ core-shell composites have good luminescence properties^16^. In short, the silica core-shell structured nanomaterials can improve its photoluminescence properties and promote their uses in biomedical applications.

Because europium ions (Eu^3+^) doped lanthanide phosphate (LaPO_4_) has unique fluorescent property, Eu^3+^-doped LaPO_4_ luminescent materials have been developed for phosphor powder, advanced flat panel displays, and biological labels^17–21^. Eu^3+^ ions used in the luminescent materials may be due to the following considerations: (1) Eu^3+^ ion has relatively high quantum efficiency. (2) The intensity ratio between electrical dipole transition (^5^D_0_ → ^7^F_2_) to magnetic dipole transition (^5^D_0_ → ^7^F_1_) transitions can be employed as a probe for the site symmetry of Eu^3+^ in the lattice. (3) The Eu^3+^ ions-doped optical material can be used as an efficient phosphor for solid state light sources^22–24^. Ray *et al*. have suggested that the local symmetry of Eu^3+^ in LaPO_4_ lattice can be determined by the intensity ratio between electrical dipole transition (^5^D_0_ → ^7^F_2_) to magnetic dipole transition (^5^D_0_ → ^7^F_1_) in different morphology nanocrystallines^25^. In addition, Jacobine *et al*. have found that the intensity ratio of the electric dipole transition to the magnetic dipole transition is higher when the products have a large fraction of Eu^3+^ ions close to the surface^26^. Thus, by means of the ratio of the electric dipole transition to the magnetic dipole transition, the effects of size, morphology, and crystallinity on the photoluminescence property of Eu^3+^ ions might be further studied.

The weak solubility of LaPO_4_ often limits its application in the field of biological fluorescence labeling. The silica surface coating may be the most effective strategy to improve the solubility, biocompatibility and the fluorescent property of LaPO_4_ samples^27,28^, because the silica shell can protect the phosphate materials from the influence of the surrounding environment. We want to develop the core-shell-shell structured SiO_2_@LaPO_4_:Eu@SiO_2_ nanomaterials, which use the LaPO_4_:Eu as the luminescent host and the SiO_2_ as cores and shells. The SiO_2_ is low cytotoxicity and inexpensiveness. Meanwhile, the SiO_2_ core can reduce the vibration of LaPO_4_:Eu molecular and enhance the photoluminescence properties of the materials. On the other hand, if the SiO_2_ were used as shells, there would be a large amount of Si-OH on the surface of the SiO_2_ shells. The Si-OH bond easily connects the nanomaterials to the biological macromolecules. Therefore, the core-shell-shell structure might protect the precious phosphors, as well as enhance the biocompatibility of the materials.

In this work, the SiO_2_ submicro-spheres were coated with layers of LaPO_4_:Eu phosphors, and the overall cost of the rare earth were reduced. Another SiO_2_ shell was coated to enhance the photoluminescence properties and the biocompatibility of the phosphate materials. The core-shell-shell structured SiO_2_@LaPO_4_:Eu@SiO_2_ submicro-spheres with different size of LaPO_4_:Eu nanoparticles were prepared to further investigate the influence of sizes on the photoluminescence properties of Eu^3+^ ions. The sizes, formation mechanism, and luminescent properties of the samples have been systemically studied. The intensity ratio of the ^5^D_0_ → ^7^F_2_ transition to the ^5^D_0_ → ^7^F_1_ transition of Eu^3+^ ions was determined. Judd-Ofelt theory was performed to evaluate the symmetry of Eu^3+^ in different-size LaPO_4_:Eu nanoparticles. Simultaneously, the photoluminescence properties of the SiO_2_@LaPO_4_:Eu@SiO_2_ submicro-spheres in aqueous solution have been investigated.

## Result and Discussion

### Structure and morphology

**Figure 1:**
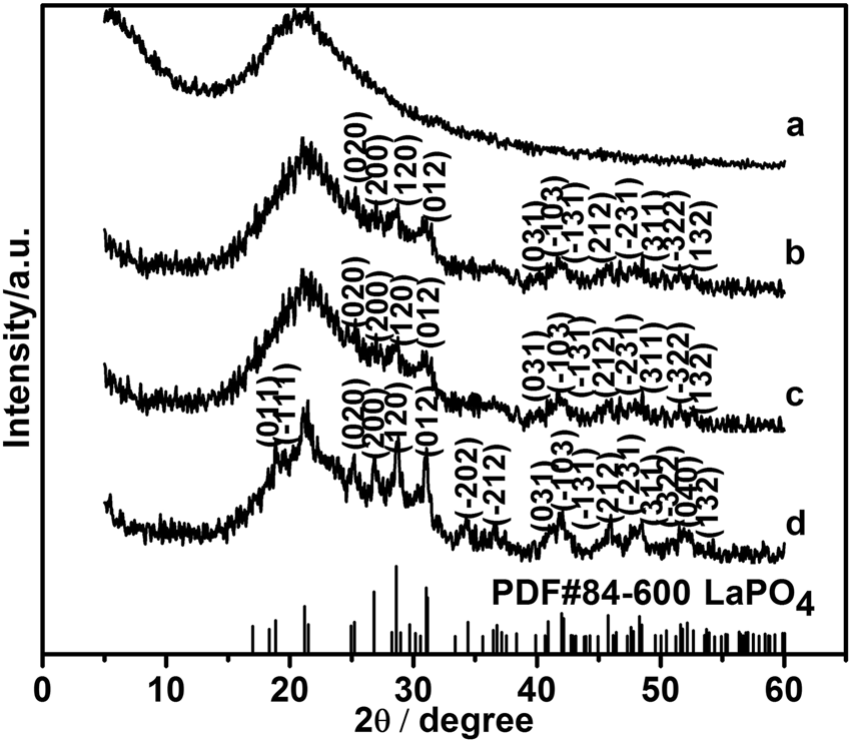
XRD patterns of SiO_2_ and core-shell-shell structured SiO_2_@LaPO_4_:Eu@SiO_2_: (**a**) SiO_2_, (**b**) S1, (**c**) S2, (**d**) S3.

When the amounts of SiO_2_ submicro-spheres were 0.200, 0.140, and 0.067 g in the reaction system, the SiO_2_@LaPO_4_:Eu@SiO_2_ submicro-spheres with different size of LaPO_4_:Eu nanoparticles were synthesized. The crystal structure of the products was identified by the XRD patterns. Figure 1 illustrates the typical XRD patterns of the products SiO_2_ submicro-spheres, S1, S2 and S3. As Fig. 1a shows, a broad band centered at 2θ = 22° from the amorphous SiO_2_ (JCPDS No. 29-0085) was observed. The diffraction peaks of S1, S2, and S3 can be attributed to the monoclinic phase of LaPO_4_ (JCPDS No. 84-600), while the broad band at 2θ = 22° might result from the amorphous SiO_2_ (Fig. 1b,c). The peaks at 18.86°, 19.62°, 25.14°, 26.90°, 28.68°, 30.94°, 34.32°, 36.76°, 40.98°, 42.08°, 42.72°, 45.92°, 47.62°, 48.52°, 51.56°, and 52.52° can be attributed to the (011), (−111), (020), (200), (120), (012), (−202), (−212), (031), (−103), (−131), (212), (−231), (311), (−322) and (132) reflections of the crystalline LaPO_4_, respectively. The results indicate that the intermediate shell LaPO_4_:Eu can be well crystallized on the surfaces of the SiO_2_ core.

**Figure 2:**
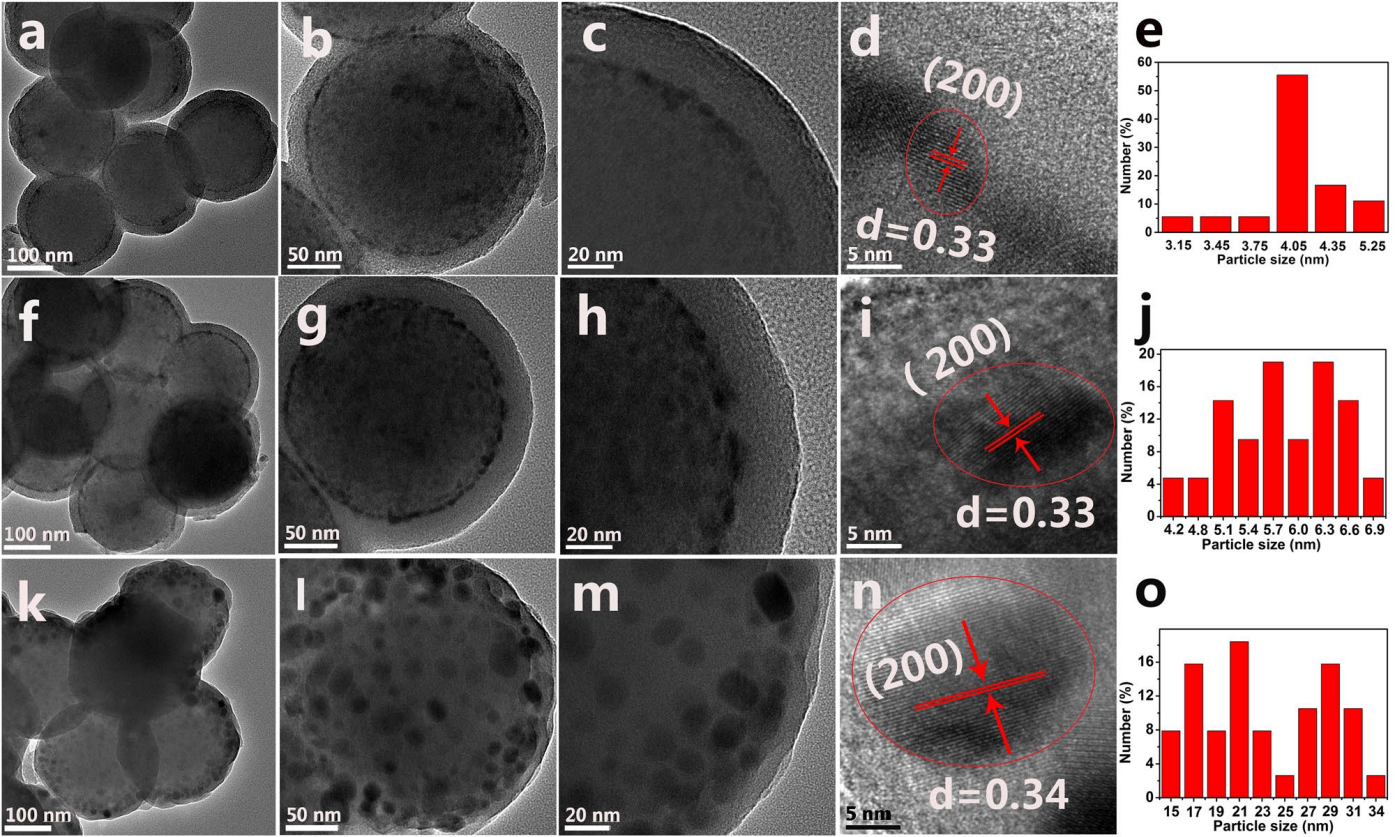
TEM, HRTEM images of the products and size distribution images of intermediate shell LaPO_4_:Eu: (**a**–**e**) S1, (**f**–**j**) S2, (**k**–**o**) S3.

The structure and morphology of the S1, S2, and S3 products were also identified by TEM and high-resolution TEM (HRTEM). Figure 2 shows the TEM and HRTEM images of the SiO_2_@LaPO_4_:Eu@SiO_2_ submicro-spheres and the particle size distribution of the intermediate shell LaPO_4_:Eu nanoparticles. Figure 2a,f,k are the low-resolution images of the products S1, S2, and S3, indicating that the three products have “core-shell-shell” structures. Moreover, the “core-shell-shell” structures were composed of uniform submicro-spheres with smooth surfaces. The high-resolution images of the products S1, S2 and S3 showed that the size of LaPO_4_:Eu nanoparticles was 4–34 nm. These nanoparticles were uniformly distributed on the surface of the SiO_2_ core, and the average diameter of the SiO_2_ core was about 200 nm (Fig. 2b,c,g,f,l,m). For the product S1, the size of the intermediate shell LaPO_4_:Eu nanoparticles was about 4 nm and the average thickness of SiO_2_ shell was about 25 nm. The size of the intermediate shell LaPO_4_:Eu nanoparticles was 5–7 nm in the product S2, and the average thickness of the SiO_2_ shell was about 25 nm. However, the intermediate shell LaPO_4_:Eu of the product S3 showed single spherical particles with 15–34 nm in size, and the average thickness of the SiO_2_ shell was about 10 nm. The HRTEM images of the products S1, S2, and S3 shows clear lattice fringes with the lattice spacing of 0.33, 0.33, and 0.34 nm, respectively. This result well agrees with the (220) crystal plane of the monoclinic phase LaPO_4_ (Fig. 2d,i,n).

**Figure 3:**
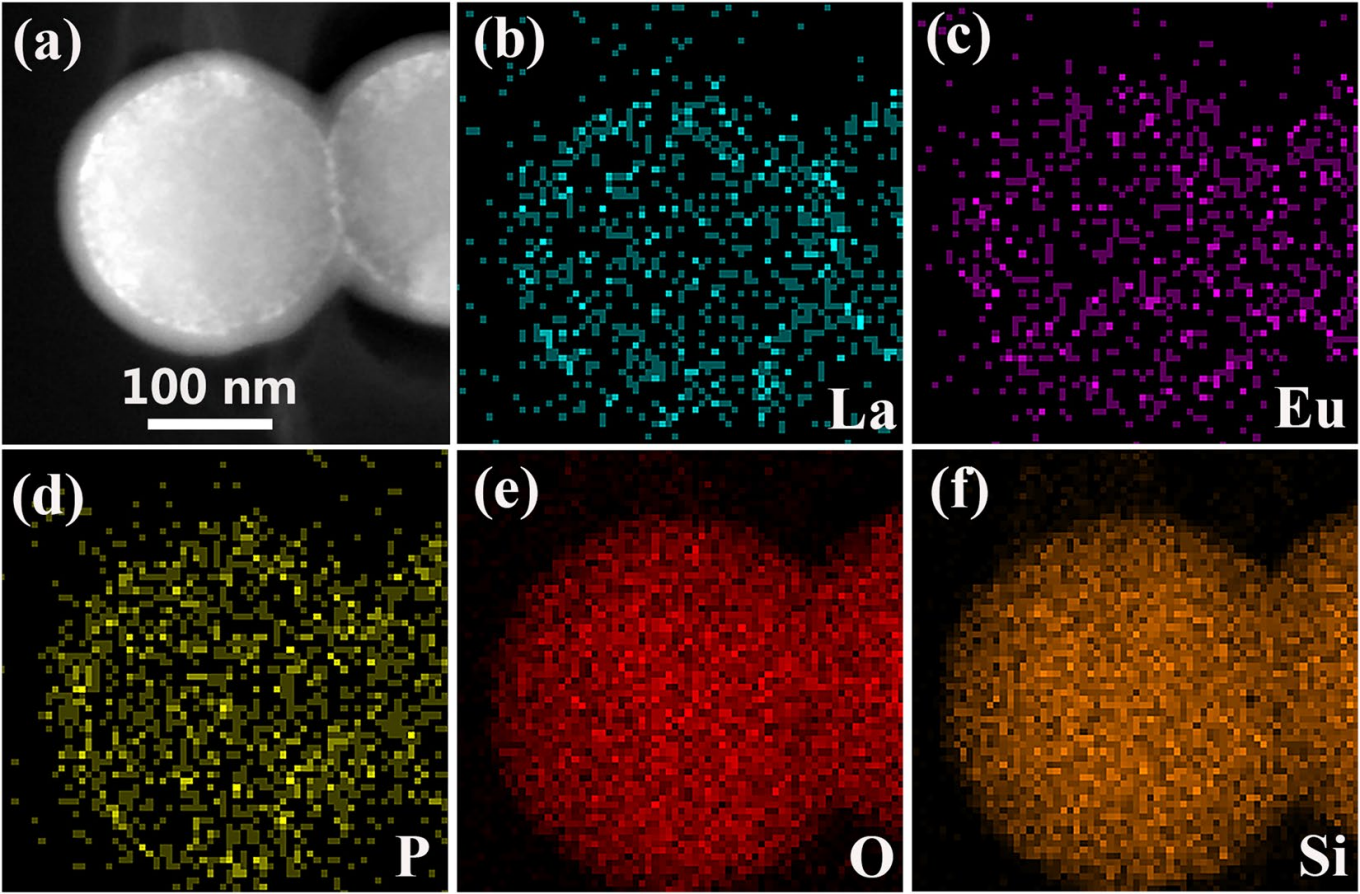
EDX mapping images of SiO_2_@LaPO_4_:Eu@SiO_2_. (**a**) STEM images, (**b**–**f**) the respective EDX element mapping images.

Figures 2e,j,o are size distribution images of the intermediate shell LaPO_4_:Eu nanoparticles in the products S1, S2, and S3, respectively. Figure 3 shows the EDX mapping image of the S1 product. The STEM image of the SiO_2_@LaPO_4_:Eu@SiO_2_ submicro-spheres indicates that the construction of the product is an obvious “core-shell-shell” structure. The elemental mapping result revealed that the La, Eu, P, O, and Si were distributed over the whole range of submicro-spheres.

To further investigate the amount of SiO_2_ submicro-spheres effects on the size of the LaPO_4_:Eu nanoparticles, the morphology of the products at the different stages were investigated by TEM (Figs S1 and S2). In this reaction system, only the amount of SiO_2_ submicro-spheres was changed and the other conditions were kept constant.

When the bridging ligand organosilane MABA-Si connected with different amount of SiO_2_ submicro-spheres, the thickness of MABA-Si grafting on the surface of SiO_2_ core was different. The thickness of the coating shell was about 2, 4, and 10 nm with decreasing the amount of SiO_2_ (Fig. S1a–l). Therefore, when different sizes of SiO_2_@MABA-Si connected with the LaPO_4_:Eu, we can obtain the SiO_2_@LaPO_4_:Eu submicro-spheres with different sizes of core-shell (the products N1, N2, and N3, Fig. S2). The TEM images (Fig. S2a–d) illustrate that LaPO_4_:Eu nanoparticles with about 4 nm in diameters could be uniformly coated on the surface of SiO_2_ submicro-spheres. By contrast, the thickness of LaPO_4_:Eu were increased to about 6 nm for the product N2 (Fig. S2e–h), and it was about 18 nm in the product N3 (Fig. S2i–l).

It is found that the thickness of SiO_2_@MABA-Si and SiO_2_@LaPO_4_:Eu would be changed by adding different amounts of SiO_2_ submicro-spheres in the reaction system. As the amount of SiO_2_ submicro-spheres decreased, we found that the thickness of SiO_2_@MABA-Si was proportionally increased. In addition, there would be higher content –COOH groups existing on the surface of the SiO_2_ submicro-spheres. The surface –COOH groups play an important role in the formation of LaPO_4_:Eu shell on the SiO_2_ core surfaces. Enough –COOH groups would coordinate with more rare earth ions, and the thickness of LaPO_4_:Eu coated on the surface of SiO_2_ core would be increased. After the core-shell-shell structured SiO_2_@LaPO_4_:Eu@SiO_2_ submicro-spheres were calcined at 900 °C, LaPO_4_:Eu particles crystallized and grew to nanoparticles with different sizes. In short, the larger thickness of the intermediate shell LaPO_4_:Eu was, the larger LaPO_4_:Eu nanoparticles might be obtained.

### The growth mechanism of SiO_2_@LaPO_4_:Eu@SiO_2_ submicro-spheres

**Figure 4:**
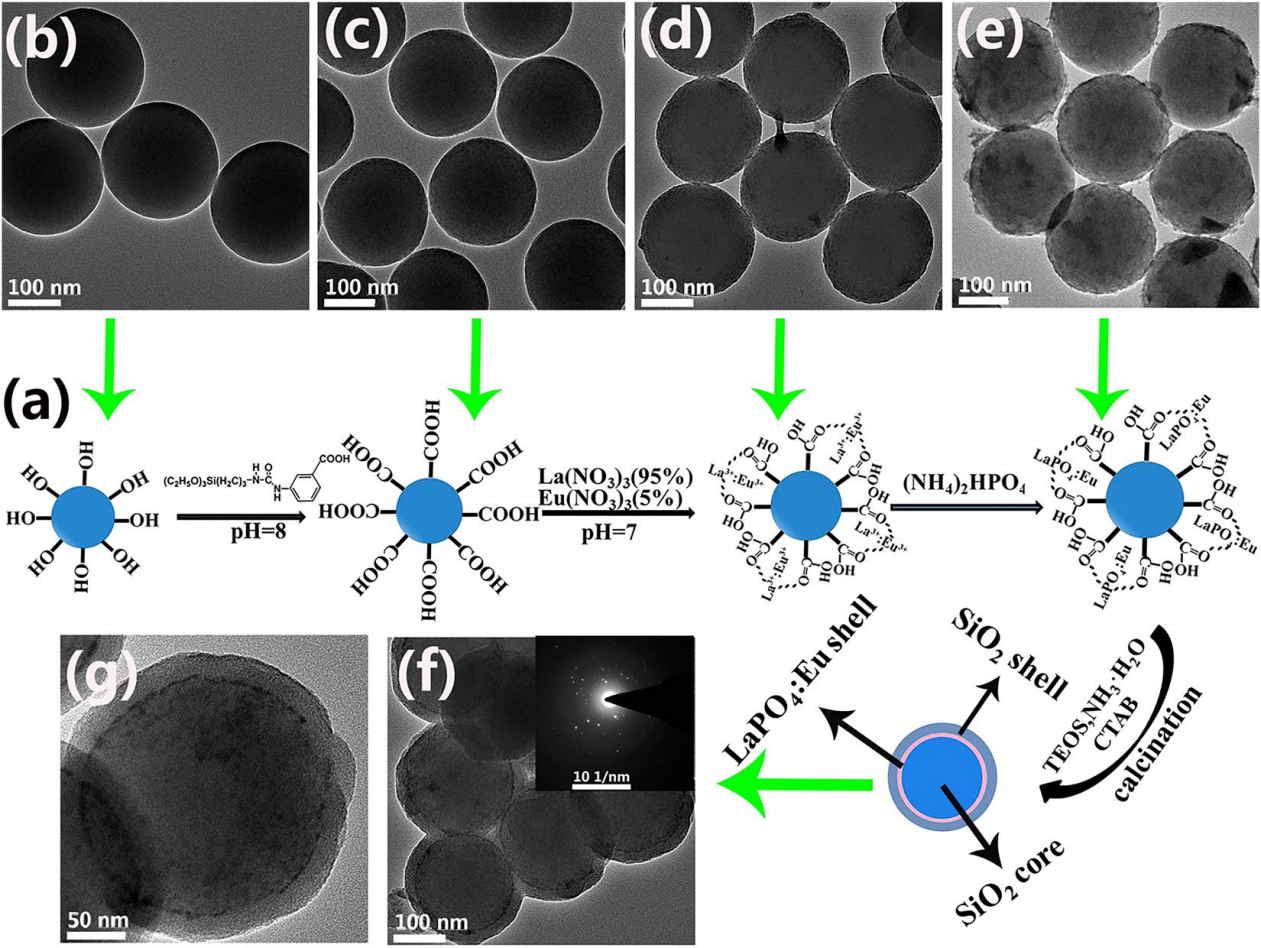
(**a**) The fabrication process of the SiO_2_@LaPO_4_:Eu@SiO_2_. TEM images of (**b**) SiO_2_, (**c**) SiO_2_@MABA-Si, (**d**) SiO_2_@La:Eu, (**e**) SiO_2_@LaPO_4_:Eu, (**f**,**g**) SiO_2_@LaPO_4_:Eu@SiO_2_ (Inset f: SAED).

**Figure 5:**
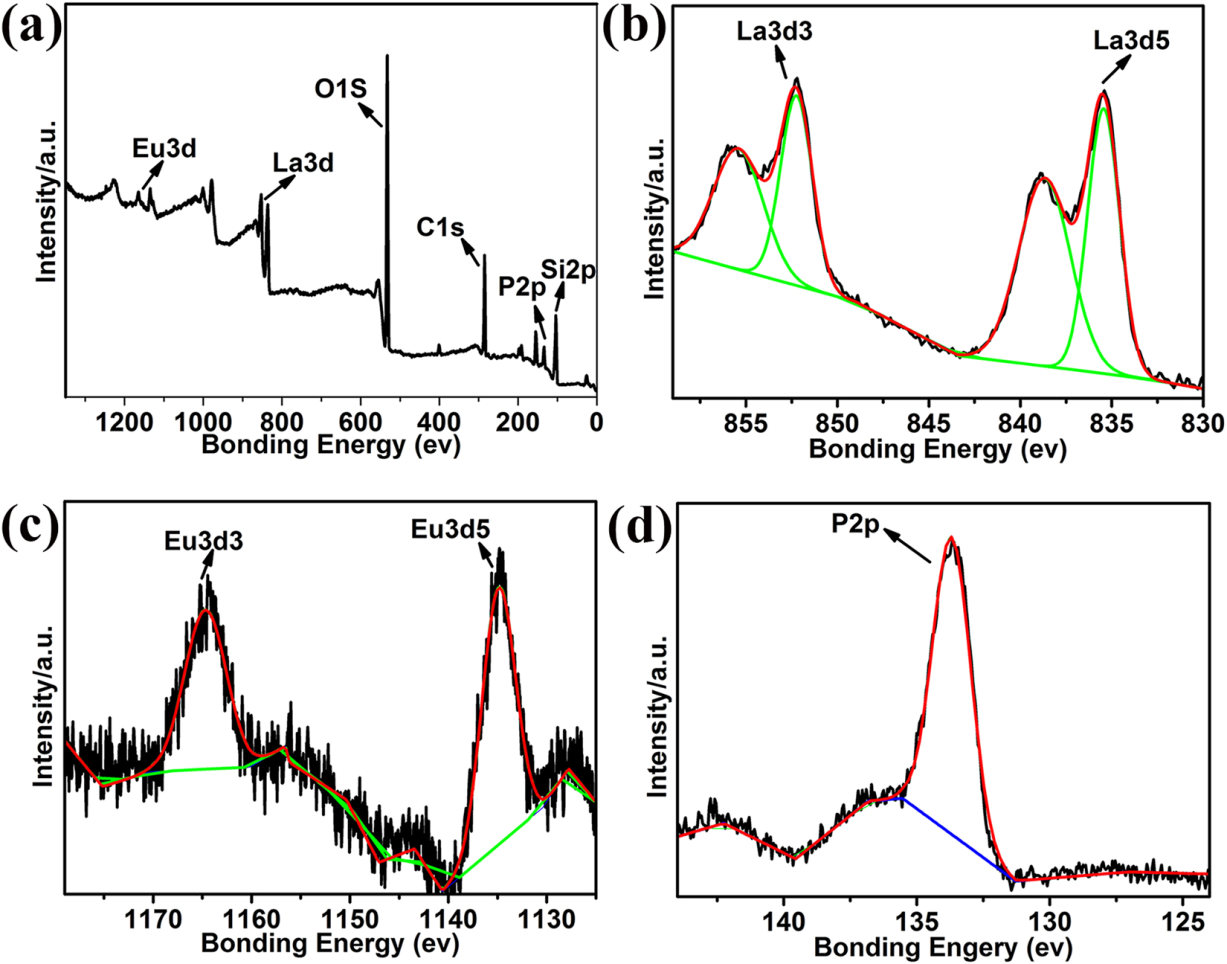
XPS spectra of SiO_2_@LaPO_4_:Eu, (**a**) survey spectrum, (**b**–**d**) high-resolution XPS spectra of La 3d, Eu 3d and P 2p.

To better understand the growth mechanism of the SiO_2_@LaPO_4_:Eu@SiO_2_ submicro-spheres, the products of S1 at different stages were studied by TEM, IR, EDX, and XPS. A possible growth mechanism for the SiO_2_@LaPO_4_:Eu@SiO_2_ submicro-spheres was proposed, as Fig. 4a–g shows. First, the SiO_2_ submicro-spheres were obtained from the hydrolysis of TEOS. The SiO_2_ submicro-spheres presented a uniform and smooth spherical morphology. The average diameter of the SiO_2_ submicro-spheres was about 200 nm (Fig. 4b). At 1104, 950, and 450 cm^−1^, the IR absorption peaks of SiO_2_ submicro-spheres are observed. They should be attributed to the vibration of Si–O–Si, Si–OH, and Si–OH stretching (Fig. S3a). Second, the bridging ligand MABA-Si was grafted on the surface of the SiO_2_ core through Si–O–Si bond that derived from the hydrolysis and the condensation of silane coupling agent. The as-synthesized SiO_2_@MABA-Si exhibited a relatively rough surface with a thin layer of about 2 nm (Fig. 4c). In the IR spectrum of SiO_2_@MABA-Si, the –COOH stretching vibrations of MABA-Si appeared at 1720 cm^−1^ (Fig. S3b). When the –COOH groups exposed onto the surface of SiO_2_ core to coordinate with La^3+^ and Eu^3+^ ions, the peak of –COOH was found at 1705 cm^−1^, which appeared an obvious red shift (Fig. S3c). As Fig. 4d shows, a shell was coated on the surface of SiO_2_ core with a thickness of about 4 nm. Third, the PO_4_^3−^ would react with rare earth ions, and the LaPO_4_:Eu nanoparticles were formed on the surface of SiO_2_@MABA-Si. It was found that the surface of submicro-spheres became rough and its thickness was about 6 nm (Fig. 4e). Furthermore, the stretching and bending vibration of PO_4_^3−^ appeared at 872 and 578 cm^−1^ in the IR spectrum of SiO_2_@LaPO_4_:Eu (Fig. S3d). EDX analysis was also used to investigate the composition of the SiO_2_@LaPO_4_:Eu (Fig. S4). The appeared peaks demonstrated that the product was composed of Si, O, P, La and Eu elements. The La and Eu elements were 19% and 9%, respectively. According to the XPS analysis results (Fig. 5), the signals of 1164, 1135, 853, 836, 134, 1300, 105, and 288 eV were assigned to the binding energies of La 3d, Eu 3d, P 2p, O 1 s, and C1s, respectively (Fig. 5a). The presence of peaks at 852 and 935 eV, associated with La elemental (Fig. 5b). The two peaks of Eu 3d were located at 1165 and 1134 eV, which was attributed to 3d^5^ and 3d^3^ (Fig. 5c). The peak of P 2p was at 134 eV (Fig. 5d). The EDX and XPS results indicated that the LaPO_4_:Eu shell structure was formed on the surface of the SiO_2_ core. Fourth, in order to form a SiO_2_ shell on the surface of SiO_2_@LaPO_4_:Eu submicro-spheres, the TEOS should be hydrolyzed slowly. After calcination, the core-shell-shell structured SiO_2_@LaPO_4_:Eu@SiO_2_ submicro-spheres were finally obtained. As shown in Fig. 4f,g, a uniform silica shell was coated onto the surface of the submicro-spheres. The SiO_2_@LaPO_4_:Eu@SiO_2_ was an obvious “core-shell-shell” structured submicro-sphere, which has a ∼25 nm outermost shell, a ∼4 nm intermediate shell, and a ∼200 nm core. The selected area electron diffraction pattern (SAED) clearly shows several diffraction points, suggesting excellent purity of the intermediate shell LaPO_4_:Eu (inset Fig. 4f). In the corresponding IR spectrum, the characteristic absorption peaks of Si–O–Si (1100 cm^−1^), the typical PO_4_^3−^ symmetrical stretching and bending vibrations (879 and 567 cm^−1^) are observed (Fig. S3e). Finally, the core-shell-shell structured SiO_2_@LaPO_4_:Eu@SiO_2_ submicro-spheres were controllably synthesized.

### Photoluminescence properties

**Figure 6:**
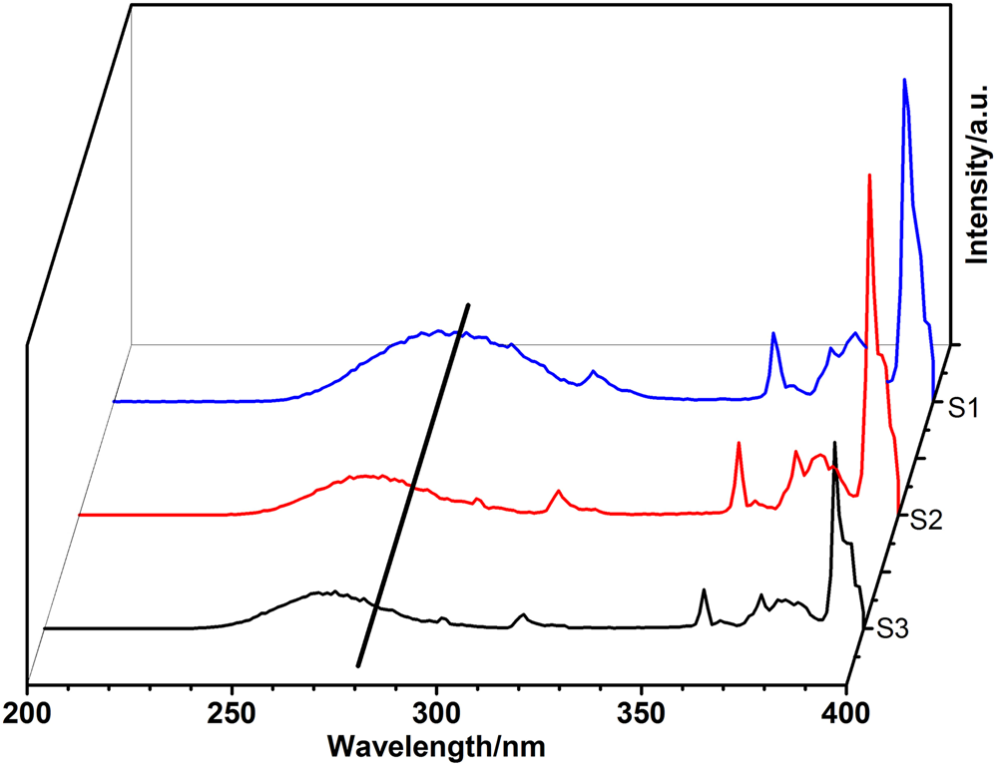
Excitation spectra of the products: S1, S2 and S3.

**Figure 7:**
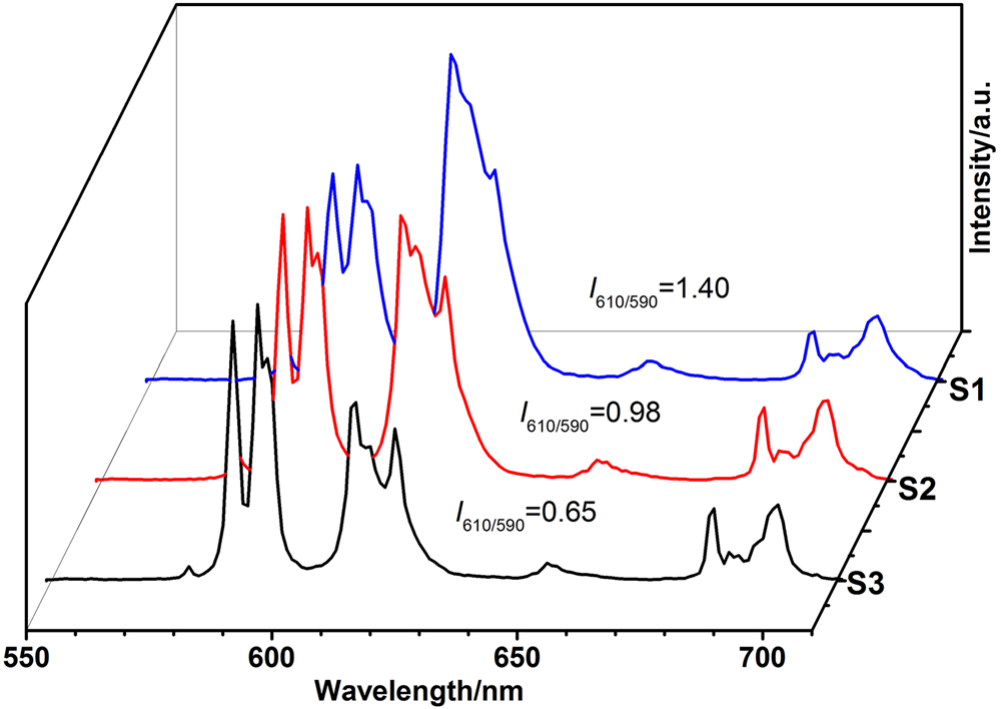
Emission spectra of the products: S1, S2 and S3.

The photoluminescence of core-shell-shell structured products with different size of LaPO_4_:Eu nanoparticles were investigated by the room-temperature photoluminescence (PL) spectra. The excitation spectra of the products S1, S2, and S3 are presented in Fig. 6. For the S1 sample, the broad band centered at 282 nm should be assigned to the charge transfer (CTB) of Eu^3+^ ions. The other four peaks at 317, 361, 375, and 393 nm are attributed to the direct excitation of the f-f shell transitions of Eu^3+^ ions^29^. The CTB for the products S2 and S3 were displayed at 271 nm and 269 nm, respectively. Generally, the CTB position depends on the Eu-O bond length. If the length of Eu-O bond is long, the CTB usually has a longer wavelength^30,31^. When the size of the LaPO_4_:Eu nanoparticles is about 4 nm, the CTB position band would show a red shift. It indicates that the Eu-O bond distance become longer and the ratio of surface Eu^3+^ ions is increased as the particle size shrinks^32^. It is found that emission peaks at 587, 612, 650, and 685 nm would excite with a 393 nm wavelength. This result should be originated from the ^5^D_0_ → ^7^F_J_ (J = 1–4) transitions of Eu^3+^ (Fig. 7)^33^. The emission peaks at 612 nm and 587 nm should correspond to the electric dipole transition ^5^D_0_ → ^7^F_2_ and the magnetic dipole transition ^5^D_0_ → ^7^F_1_ of Eu^3+^ ions. The S1–S3 samples have the same peak positions in the emission spectra. However, the intensity patterns of these products are different. The strongest peak for the S1 product is found at 612 nm, but at 587 nm for the products S2 and S3. Additionally, the intensity ratios of the electric dipole transition ^5^D_0_ → ^7^F_2_ to magnetic dipole transition ^5^D_0_ → ^7^F_1_ in the products are different, and the calculated intensity ratios are 1.40, 0.98 and 0.45 for the products S1, S2, and S3, respectively. With the size of the LaPO_4_:Eu nanoparticles decreases, the intensity ratios of the ^5^D_0_ → ^7^F_2_ transition to the ^5^D_0_ → ^7^F_1_ transition increases gradually, and the intensity of the electric dipole transition ^5^D_0_ → ^7^F_2_ becomes stronger. These results indicate that the PL properties depend on the size of LaPO_4_:Eu nanoparticles. Generally, the electric-dipole transitions are strictly forbidden, and the magnetic-dipole transitions are permitted due to the parity selection rules. The electric-dipole transition ^5^D_0_ → ^7^F_2_ is very sensitive to the local environment^34^. When the Eu^3+^ ions do not lie on an inversion center of the crystal, the forbiddance of electric-dipole transition is resolved to some extent, and the electric-dipole transition ^5^D_0_ → ^7^F_2_ may become stronger. It is believed that the crystal field around the Eu^3+^ ions should not much affect the magnetic dipole transition ^5^D_0_ → ^7^F_1_^35^. When the size of the nanoparticles decreased, the ratio of Eu^3+^ ions in the surface of nanoparticles would be increased. Therefore, the decrease of the symmetry around the Eu^3+^ ions would lead to the enhancement of the ^5^D_0_ → ^7^F_2_ transition^32,36,37^. To further understand the f-f transition and the local symmetry properties of Eu^3+^ ions in the LaPO_4_ crystal lattice, the optical transition strength parameters (Ω_2_ and Ω_4_) were calculated by the well-known Judd-Ofelt (J-O) theory.

The Judd-Ofelt (J-O) theory describes f-f transition properties of rare earth ions and realizes the parameterization of optical transition strength and transition probability^38,39^. Thus, according to the emission spectra and the J-O theory, the optical transition strength parameters (Ω_2_ and Ω_4_) can be calculated. A higher Ω_2_ value means a decrease in site symmetry. The variation of the Ω_2_ value often relate to the change in local symmetry around the Eu^3+^ ions due to the hypersensitivity of the electric dipole transition ^5^D_0_ → ^7^F_2_ to the local environment^40^. Here, in order to further understand the local symmetry properties of the Eu^3+^ ions, J-O theory was used to calculate Ω_2_ and Ω_4_ by analyzing the emission spectrum of the products S1–S3. As the J-O theory formula (1) shows, the transition rate of the energy level is in proportion with the integral strength of the emission spectrum. Thus, the magnetic dipole transition ^5^D_0_ → ^7^F_1_ of Eu^3+^ ion is independent of the environment and can be used as a reference^41^. We can calculate Ω_2_ and Ω_4_ values by calculating the integrated intensity of the electric-dipole transitions ^5^D_0_ → ^7^F_2_ and ^5^D_0_ → ^7^F_4_. The ratio of the electric dipole transition rate to magnetic dipole transition rate can be expressed as:

1 $$\frac{{{\rm{A}}}_{0{\rm{J}}}}{{{\rm{A}}}_{01}}=\frac{{{\rm{e}}}^{2}}{{{\rm{S}}}_{{\rm{md}}}}\frac{{{\rm{v}}}_{{\rm{J}}}^{3}}{{{\rm{v}}}_{{\rm{1}}}^{3}}\frac{{({{\rm{n}}}^{2}+2)}^{2}}{9{{\rm{n}}}^{2}}\,{\Omega }_{{\rm{\lambda }}}|\langle {}^{5}{\rm{D}}_{0}\Vert {{\rm{U}}}^{(\lambda )}\Vert {{}^{7}{\rm{F}}_{{\rm{J}}}\rangle |}^{2}=\frac{\int {{\rm{I}}}_{{\rm{J}}}{\rm{dv}}}{\int {{\rm{I}}}_{{\rm{1}}}{\rm{dv}}}$$

where, A_01_ refers to magnetic dipole transition rates. It is independent of the environment and has a defined value of 50 s^−1^ ^42^. The magnetic dipole transition rates A_01_ is expressed as:

2 $${{\rm{A}}}_{01}=\frac{64{{\rm{\pi }}}^{4}{{\rm{v}}}_{1}^{3}{{\rm{n}}}^{3}{{\rm{S}}}_{{\rm{md}}}}{3{\rm{h}}(2{\rm{J}}+1)}$$

The electric dipole transition rates A_0J_ is expressed as:

3 $${{\rm{A}}}_{0{\rm{J}}}=\frac{64{{\rm{\pi }}}^{4}{{\rm{v}}}_{{\rm{J}}}^{3}}{3{\rm{h}}(2{\rm{J}}+1)}{{\rm{e}}}^{2}\frac{{\rm{n}}{({{\rm{n}}}^{2}+2)}^{2}}{9}\sum {\Omega }_{{\rm{\lambda }}}|\langle {}^{{\rm{5}}}{\rm{D}}_{0}\Vert {{\rm{U}}}^{({\rm{\lambda }})}\Vert {{}^{7}{\rm{F}}_{{\rm{J}}}\rangle |}^{2}$$

e is the electronic charge and the value is 4.803 × 10^−10^; S_md_ denotes the magnetic dipole line strength and its value is a constant and independent of the host materials. The value of S_md_ is 9.6 × 10^−42^ units^43^; h is Planck’s constant and its value is 6.626 × 10^−27^; n is the refractive index of the phosphors and its value is 1.6; v_1_ and ν_J_ are the wavenumbers of the corresponding transition.

$|\langle {}^{5}{\rm{D}}_{0}|{{\rm{U}}}^{({\rm{\lambda }})}|{}^{7}{\rm{F}}_{{\rm{J}}}\rangle |$ is the squared reduced lattice element. Their values are also independent of the environment of Eu^3+^ and are 0.00324 and 0.00229 for J = 2 and 4, respectively^44^.

$\frac{\int {{\rm{I}}}_{{\rm{J}}}{\rm{dv}}}{\int {{\rm{I}}}_{1}{\rm{dv}}}$ can be obtained from the integrated intensity corresponding to the ^5^D_0_ → ^7^F_1_ and ^5^D_0_ → ^7^F_J_ (J = 2, 4) transitions in the emission spectra. For the products S1, S2, and S3, we calculated the value of $\frac{\int {{\rm{I}}}_{2}{\rm{dv}}}{\int {{\rm{I}}}_{1}{\rm{dv}}}$ is 1.77, 1.38, and 0.95. The value of $\frac{\int {{\rm{I}}}_{4}{\rm{dv}}}{\int {{\rm{I}}}_{1}{\rm{dv}}}$ is 0.37, 0.40 and 0.39, respectively. Substituting the $\frac{\int {{\rm{I}}}_{2}{\rm{dv}}}{\int {{\rm{I}}}_{1}{\rm{dv}}}$ values of the products S1, S2, and S3 into the formula (1), we can get the value of Ω_2_ is 2.30 × 10^−20^, 1.80 × 10^−20^, and 1.20 × 10^−20^. Substituting the $\frac{\int {{\rm{I}}}_{4}{\rm{dv}}}{\int {{\rm{I}}}_{1}{\rm{dv}}}$ values of the products S1, S2 and S3 into the formula (1), we can get the value of Ω_4_ is 1.00 × 10^−20^, 1.08 × 10^−20^, and 1.05 × 10^−20^. The calculation results are shown in Table 1.

**Table 1:** The J-O parameters of SiO_2_@LaPO_4_:Eu@SiO_2_.

Size of LaPO_4_:Eu, nm	*A*_01_, s^−1^	*A*_02_, s^−1^	*A*_04_, s^−1^	*A*_rad_, s^−1^	Ω_2_, × 10^−20^ cm^2^	Ω_4_, × 10^−20^ cm^2^	R
4	50	88.52	18.54	162.06	2.30	1.00	1.77
5~7	50	69.05	20.03	143.45	1.80	1.08	1.38
15~34	50	47.51	19.52	120.48	1.20	1.05	0.95

In our study, when the size of LaPO_4_:Eu nanoparticles is about 4 nm (Ω_2_ is 2.30 × 10^−20^), the intensity ratio of the ^5^D_0_ → ^7^F_2_ to ^5^D_0_ → ^7^F_1_ is greater than 1.0. This result indicates that the excited RE^3+^ was not in the symmetric center of the lattice. Moreover, the result also shows that the forbiddance of electric-dipole transition was resolved to some extent because of the perturbation of the crystal field. When the size of LaPO_4_:Eu nanoparticles is 5–34 nm (Ω_2_ ≤ 1.80 × 10^−20^), the intensity ratios of the ^5^D_0_ → ^7^F_2_ to ^5^D_0_ → ^7^F_1_ transitions is less than 1.0, indicating that the Eu^3+^ ion locates in the symmetric center of the LaPO_4_ lattice.

The results were consistent with the CIE chromaticity diagram of the products S1, S2, and S3, which were estimated from their emission spectra (Fig. S5). The CIE chromaticity coordinates of the product S1 are closer to the red light than those of the product S2 or S3. Moreover, the parameter Ω_4_ was hardly affected by the symmetry of the Eu^3+^ ions in the LaPO_4_ lattice^45^. The relative intensity ratio (R) of ^5^D_0_ → ^7^F_2_ to ^5^D_0_ → ^7^F_1_ can be calculated by the formula (4). According to the emission spectra of the products, we calculated the R values of the S1–3 products. When the size of the nanoparticles decreased, the value of R would increase, and the symmetry of the Eu^3+^ ions in the LaPO_4_ lattice was decreased (Table 1).

4 $${\rm{R}}=\frac{{\int }_{602}^{637}{{\rm{I}}}_{0-2}d{\rm{\lambda }}}{{\int }_{581}^{602}{{\rm{I}}}_{0-1}d{\rm{\lambda }}}$$

The photoluminescence lifetime of the products with different sizes of LaPO_4_:Eu nanoparticles was also measured. The photoluminescence fitting curves of the products S1, S2, and S3 were shown in Fig S6. The calculated average lifetimes (τ) were calculated to be 1.53, 2.99, and 1.17 ms for the products S1, S2, and S3, respectively. Simultaneously, the absolute quantum yields were 40.23%, 11.26%, and 11.68% for the products S1, S2, and S3, respectively.

**Figure 8:**
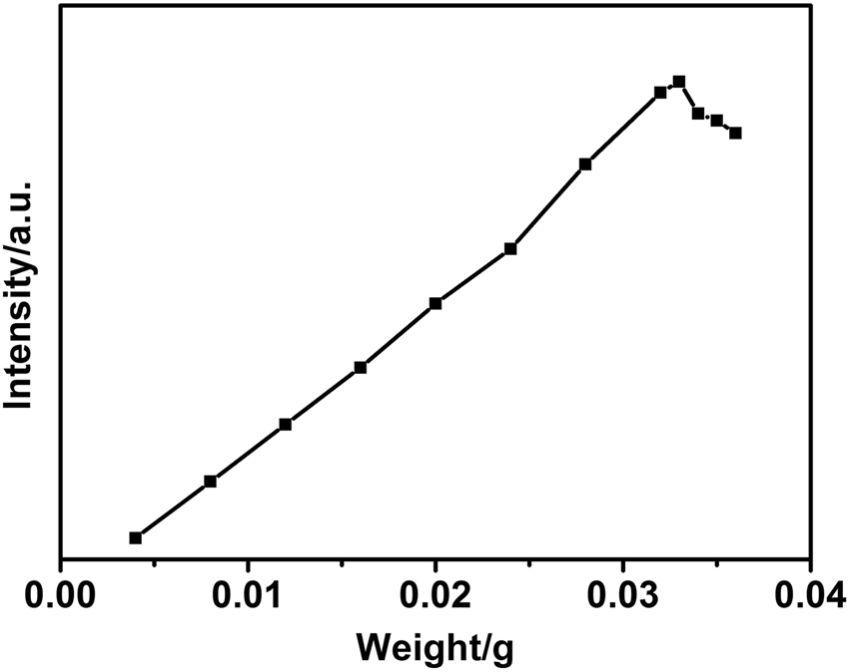
Emission intensity of the product S1 in 5 mL H_2_O as a function of weight.

**Figure 9:**
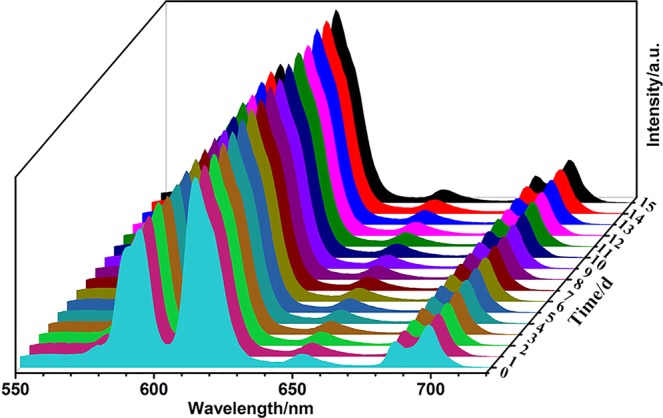
Emission spectra of 0.033 g the product S1 in 5 mL H_2_O with various times.

In order to investigate the possibility of SiO_2_@LaPO_4_:Eu@SiO_2_ submicro-spheres in the biological application, we studied the relation of the concentration, the placement time, and the PL properties of SiO_2_@LaPO_4_:Eu@SiO_2_ submicro-spheres in aqueous solution. Figure 8 displays the emission intensity *vs*. the concentration of S1 in 5 mL H_2_O. At first, the emission intensity of SiO_2_@LaPO_4_:Eu@SiO_2_ would increase with the increase of the concentration, but it comes to stable at last. When 5 mL H_2_O contained 0.033 g product S1, the strongest emission intensity could be detected. Because of the rate –OH quenching effect, the PL emission intensity in water gives a slight decrease with respect to that in the solid state. However, the SiO_2_@LaPO_4_:Eu@SiO_2_ submicro-spheres in aqueous solution shows a stable emission property. As Fig. 9 shows, the emission of SiO_2_@LaPO_4_:Eu@SiO_2_ would not be quenched even after 15 days. This good photoluminescence stability in aqueous solution might offer many opportunities for their applications in fluorescent bio-labeling/bioimaging and drug delivery.

## Conclusions

In summary, the core-shell-shell structured rare earth phosphates luminescent materials (SiO_2_@LaPO_4_:Eu@SiO_2_) were controllably synthesized by a simple co-precipitation method using silane coupling agent (MABA-Si). The SiO_2_ shell played a key role in perfecting the solubility and improving the photoluminescence properties of the products. By varying the thickness of MABA-Si grafting on the SiO_2_ core and selecting the appropriate substitution reaction of phosphate, the intermediate shell LaPO_4_:Eu nanoparticles with different sizes can be obtained. According to the PL spectra and the calculation results of the Judd-Ofelt theory, the size of LaPO_4_:Eu significantly impacts on the symmetry of Eu^3+^ ions in LaPO_4_ lattice. When the size of LaPO_4_:Eu nanoparticles was about 4 nm (Ω_2_ = 2.30 × 10^−20^), the symmetry of Eu^3+^ ions in the crystal field became lower. Simultaneously, the SiO_2_@LaPO_4_:Eu@SiO_2_ exhibited strong red luminescence, which would correspond to the ^5^D_0_ → ^7^F_2_ transition of the Eu^3+^ ions. If the sizes of LaPO_4_:Eu nanoparticles were 5–34 nm (Ω_2_ ≤ 1.80 × 10^−20^), the Eu^3+^ ion would locate in the symmetric center of the LaPO_4_ lattice. Even over 15 days, the PL emission intensity of SiO_2_@LaPO_4_:Eu@SiO_2_ was stable in aqueous solution. These studies might expand the application of submicro-spheres in the field of the fluorescent bio-label/bio-image.

## Materials and Methods

### Reagents

Eu_2_O_3_ (99.99%), La(NO_3_)_3_·6H_2_O, Tetraethoxysilane (TEOS), Ammonia, Cetyltrimethyl ammonium bromide (CTAB), and (NH_4_)_2_HPO_4_ were purchased from Sinopharm Chemical Reagent Beijing Corporation Limited. Aladdin (Shanghai, China) provided 3-(triethoxysilyl)-propyl isocyanate and m-aminobenzoic acid. All reagents were analytical grade without further purification. The europium nitrate powder was prepared from Eu_2_O_3_. It was further dissolved in 10% nitric acid and was dried in a vacuum box.

### Synthesis of SiO_2_@LaPO_4_:Eu@SiO_2_ submicro-spheres

The SiO_2_@LaPO_4_:Eu@SiO_2_ submicro-spheres are core-shell-shell structures, which were synthesized according to our earlier report^46^. First, the synthesis progress of SiO_2_@LaPO_4_:Eu was briefly described as follows: (1) The Stöber method was employed to synthesize the SiO_2_ submicro-spheres^47^. (2) The MABA-Si (bridging ligand organosilane) was prepared by using the method reported in literature^48^. (^1^H NMR, δ ppm: (2H) 0.56, (9H) 1.04–1.15, (2H) 2.93, (2H) 3.06, (6H) 3.33–3.75, (1H) 6.20, (4H) 7.43–8.01, (1H) 8.65 and (1H) 12.88). (3) The as-synthesized MABA-Si (1.000 g) and SiO_2_ submicro-spheres (0.067–0.200 g) were mixed with 25 mL ethanol. The pH value of the mixture was adjusted to ~8.0 by adding ammonia water, and the solution was magnetic-stirred for 4 h. The obtained SiO_2_@MABA-Si solution should be washed by ethanol three times. The obtained depositions were dispersed in 10 mL ethanol followed by a slowly addition of RE(NO_3_)_3_ (95% La^3+^ and 5% Eu^3+^) ethanol solution (0.066 mol/L) under stirring for 4 h. Finally, the suitable (NH_4_)_2_HPO_4_ was added into the mixture under continuous stirring for 6 h and a precipitation of core-shell structured SiO_2_@LaPO_4_:Eu submicro-spheres were synthesized. The materials were further washed by water and ethanol, and were dried under air at 60 °C for 8 h. The details of obtained core-shell structured SiO_2_@LaPO_4_:Eu submicro-spheres were displayed in Table 2.

**Table 2:** Prepared conditions of the core-shell structured SiO_2_@LaPO_4_:Eu submicro-spheres.

Products number	MABA-Si, (g)	SiO_2_ core, (nm)	SiO_2_ core, (g)	LaPO_4_:Eu thickness, (nm)
N1	1.000	~200	0.200	~4
N2	1.000	~200	0.140	~6
N3	1.000	~200	0.067	~18

Second, the synthesis of the SiO_2_@LaPO_4_:Eu@SiO_2_ submicro-spheres can be described as following. The above synthesized core-shell structured SiO_2_@LaPO_4_:Eu submicro-spheres (N1, N2, or N3, 0.100 g) were dispersed into 20 mL mixture solution of deionized water and ethanol. Subsequently, 0.1 g CTAB was introduced into the above mixture followed by dripping 1.0 mL aqueous ammonia with a concentration of 2.0 mol·dm^−3^. The suitable tetraethoxysilane (TEOS) was dropwise added to this solution and magnetic-stirred for 6 h. When the white solid precipitation was found, it was filtered and dried at 60 °C for 8 h. Finally, the above products synthesized from N1, N2, and N3 were further calcined in muffle furnace at 900 °C for 4 h, which were defined as S1, S2, and S3, respectively.

### Characterization

The scanning electronic microscopy (SEM; Hitachi S-4800, Japan) and the transmission electron microscopy (TEM; FEI Tecnai F20, USA) were used to characterize the structure and morphology of the products. In addition, XRD data were investigated by a X-ray diffractometer (Model M21XVHF22, MAC science Co. Ltd., Japan). The characterization was carried out by using Cu Kα radiation over a 2θ range of 10–60° at room temperature. The X-ray photoelectron spectrometer (XPS, Thermo ESCALab 250Xi, USA) was also used to identify the elemental valences of the sample. The Infrared spectra of powders were recorded on a FT-IR instrument (IR, Nicolet NEXUS 670, USA) with a range of 4000–400 cm^−1^. At room temperature, the photoluminescence of the samples were determined on a fluorescence photometer (FL; Edinburgh S980, UK). And the quantum yields of products were measured at solid state (FL; Edinburgh S980, UK).

## Supplementary information

Supplemental file
