# Supplementary material for: Luminescence Studies and Judd–Ofelt Analysis on SiO2@LaPO4:Eu@SiO2 Submicro-spheres with Different Size of Intermediate Shells
Source: Sci Rep. 2019 Sep 10;9:13065. doi: 10.1038/s41598-019-49323-6 (PMC6737155; doi:10.1038/s41598-019-49323-6)
Supplement: Supplementary file 1 — Supplemental file [file 41598_2019_49323_MOESM1_ESM.docx]

**Luminescence Studies and Judd–Ofelt Analysis on SiO_2_@LaPO_4_:Eu@SiO_2_ Submicro-spheres with Different Size of Intermediate Shells**

**Xiaowei Zhu**^1^**, Kuisuo Yang**^2^**, Anping Wu**^2^**, He Bai**^2^**, Jinrong Bao** ^2*^**, Yan Qiao**^2^**, Yunjiang Yang**^2^**, Wenxian Li**^2^**, Ying Liu**^2^

^1^ College of Pharmacology, Inner Mongolia Medical University, Hohhot 010059, China,

^2^ Inner Mongolia Key Laboratory of Chemistry and Physics of Rare Earth Materials, College of Chemistry and Chemical Engineering, Inner Mongolia University, Hohhot 010021, China,

^*^ To whom the correspondence should be addressed. Tel: (+86)-0471-4992981. E-Mail: jinrongbao@imu.edu.cn (J. R. Bao).


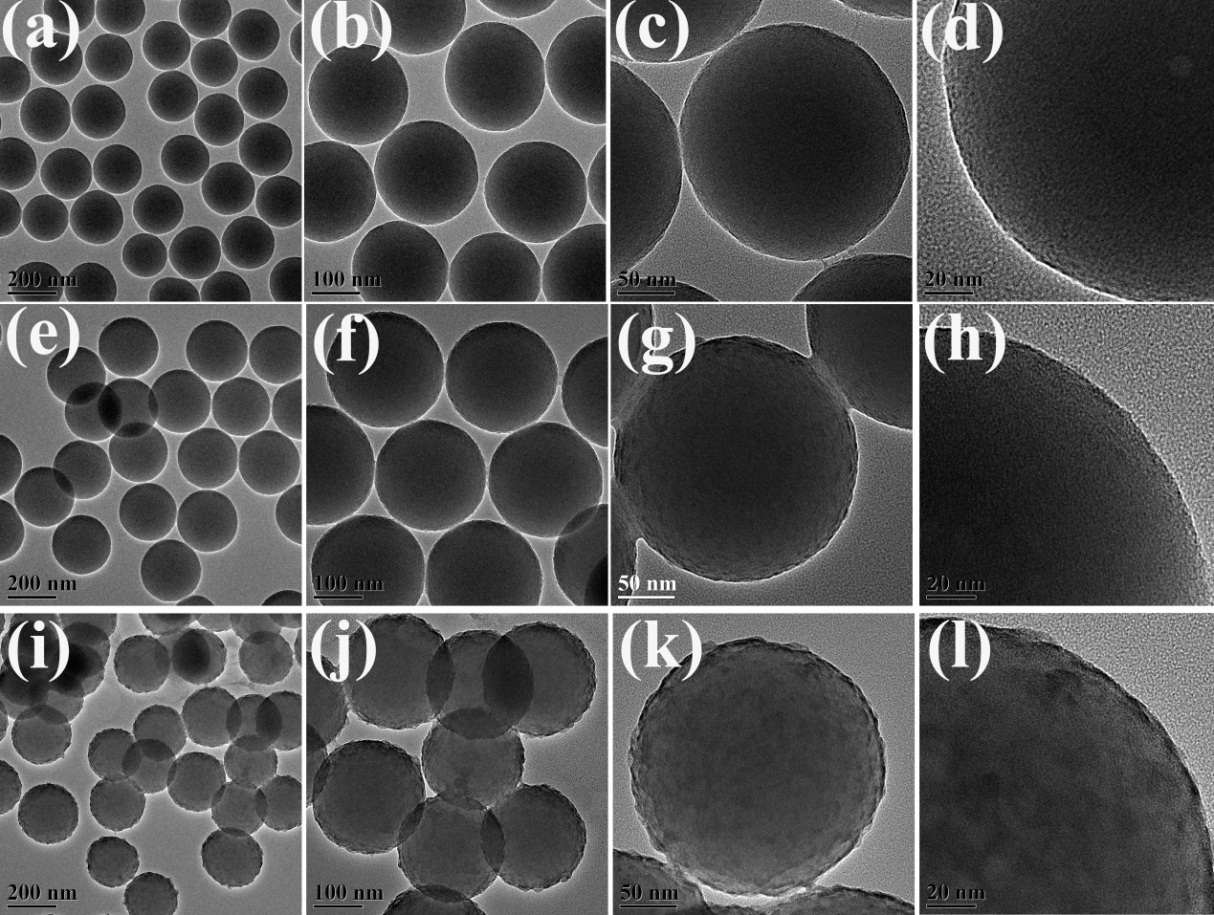


**Supplementary Figure S1.** TEM images of the middle products SiO_2_@MABA-Si prepared with different amount of SiO_2_ submicro-spheres: (a-d) 0.200 g, (e-h) 0.140 g and (i-l) 0.067 g.


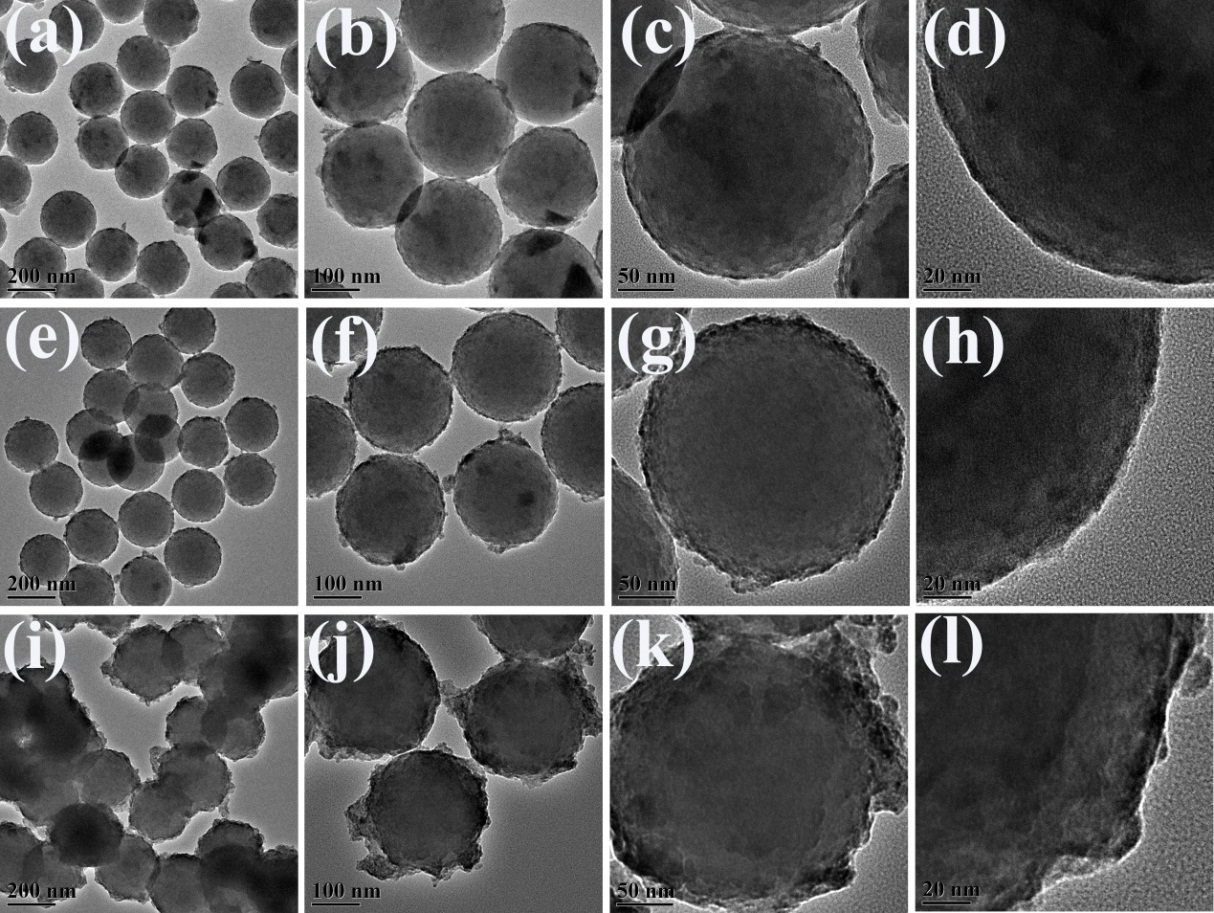


**Supplementary Figure S2.** TEM images of the middle products SiO_2_@LaPO_4_:Eu: N1 (a-d), N2 (e-h) and N3 (i-l).


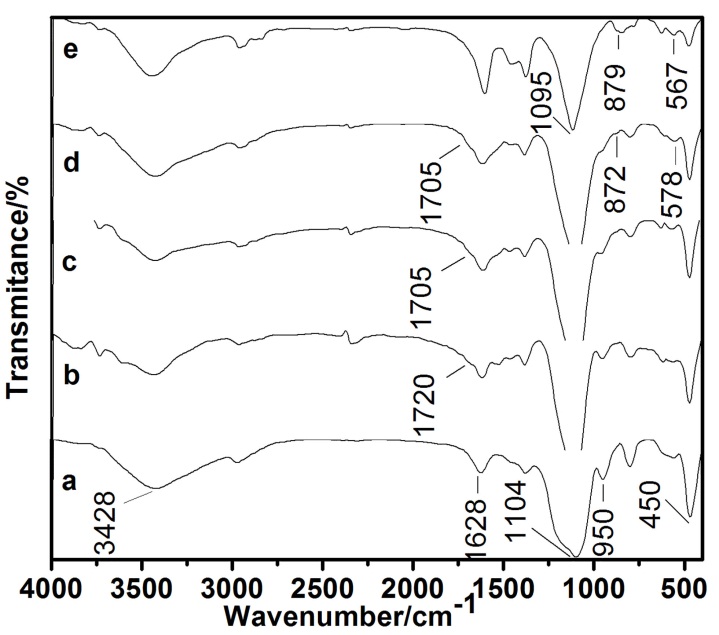


**Supplementary Figure S3.** IR spectra of product S1 prepared at various stages: (a) SiO_2_, (b) SiO_2_@MABA-Si, (c) SiO_2_@La:Eu, (d) SiO_2_@LaPO_4_:Eu, (e) SiO_2_@LaPO_4_:Eu@SiO_2_.


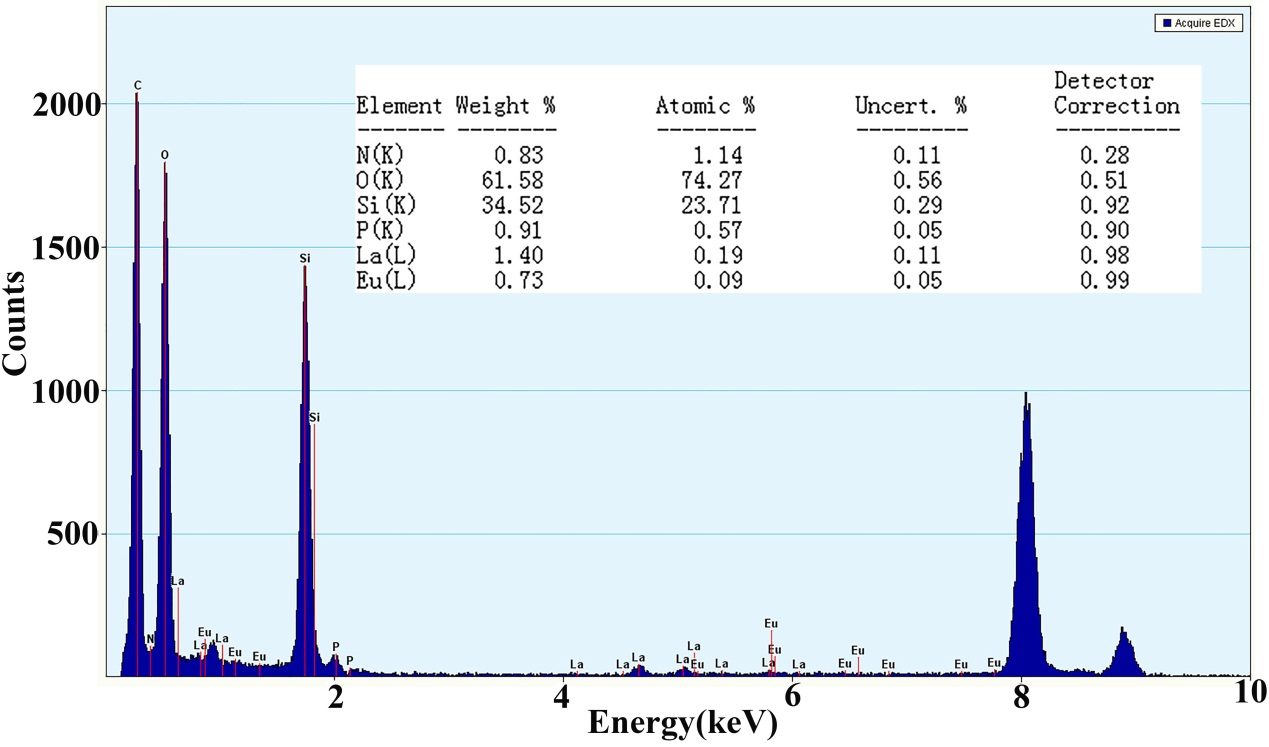


**Supplementary Figure S4.** EDX spectrum of SiO_2_@LaPO_4_:Eu


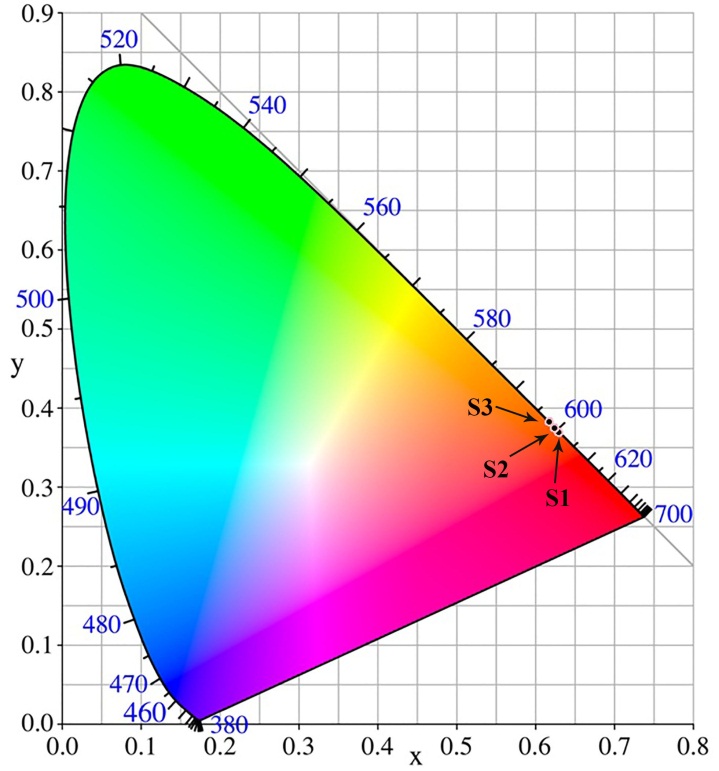


**Supplementary Figure S5.** CIE chromaticity diagram of the products S1, S2 and S3.


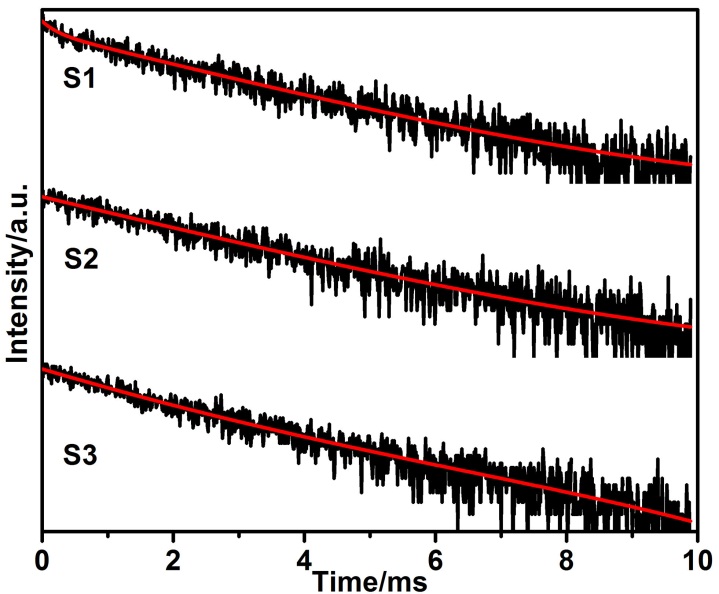


Supplementary Figure S6. The photoluminescence fitting curves of the products: S1, S2, and S3.
